# Protective Role of Flavonoids against Intestinal Pro-Inflammatory Effects of Silver Nanoparticles

**DOI:** 10.3390/molecules26216610

**Published:** 2021-10-31

**Authors:** Ana T. Rufino, Ana Ramalho, Adelaide Sousa, José Miguel P. Ferreira de Oliveira, Paulo Freitas, Manuel A. Gonzalez Gómez, Yolanda Piñeiro-Redondo, José Rivas, Félix Carvalho, Eduarda Fernandes, Marisa Freitas

**Affiliations:** 1 LAQV, REQUIMTE, Laboratory of Applied Chemistry, Department of Chemical Sciences, Faculty of Pharmacy, University of Porto, 4050-313 Porto, Portugal; ana.t.rufino@gmail.com (A.T.R.); analeileiana@gmail.com (A.R.); adelaidetsousa@gmail.com (A.S.); jmoliveira@ff.up.pt (J.M.P.F.d.O.); 2 International Iberian Nanotechnology Laboratory, 4715-330 Braga, Portugal; paulo.freitas@inl.int; 3 Nanotechnology and Magnetism Lab—NANOMAG, Department of Applied Physics, Universidade de Santiago de Compostela, 15782 Santiago de Compostela, Spain; manuelantonio.gonzalez@usc.es (M.A.G.G.); y.pineiro.redondo@usc.es (Y.P.-R.); jose.rivas@usc.es (J.R.); 4 UCIBIO, REQUIMTE, Laboratory of Toxicology, Department of Biological Sciences, Faculty of Pharmacy, University of Porto, 4050-313 Porto, Portugal; felixdc@ff.up.pt

**Keywords:** inflammation, nanoparticles, intestinal cells, neutrophils, flavonoids

## Abstract

Silver nanoparticles (AgNP) have been increasingly incorporated into food-related and hygiene products for their unique antimicrobial and preservative properties. The consequent oral exposure may then result in unpredicted harmful effects in the gastrointestinal tract (GIT), which should be considered in the risk assessment and risk management of these materials. In the present study, the toxic effects of polyethyleneimine (PEI)-coated AgNP (4 and 19 nm) were evaluated in GIT-relevant cells (Caco-2 cell line as a model of human intestinal cells, and neutrophils as a model of the intestinal inflammatory response). This study also evaluated the putative protective action of dietary flavonoids against such harmful effects. The obtained results showed that AgNP of 4 and 19 nm effectively induced Caco-2 cell death by apoptosis with concomitant production of nitric oxide, irrespective of the size. It was also observed that AgNP induced human neutrophil oxidative burst. Interestingly, some flavonoids, namely quercetin and quercetagetin, prevented the deleterious effects of AgNP in both cell types. Overall, the data of the present study provide a first insight into the promising protective role of flavonoids against the potentially toxic effects of AgNP at the intestinal level.

## 1. Introduction

The growing developments in the nanoscience and nanotechnology fields have resulted in several consumer products, many of which are routinely used in our daily life. Among the 1814 products listed in the Nanotechnology Consumer Products Inventory, 438 (24%) contain silver nanoparticles (AgNP) [1]. Due to their unique antimicrobial properties, AgNP were incorporated in all phases of food production process (processing, packaging and storage). Therefore, human dietary intake of AgNP may result in an extensive exposure of the human gastrointestinal tract (GIT) to these nanoparticles, raising questions about potentially deleterious health effects at this level [2].

Despite the controversial data on AgNP cytotoxicity, varying from absence of cytotoxicity [3], to time-, size- and dose-dependent cytotoxicity [4,5,6], literature also reports that AgNP suspensions release silver ions that may contribute to harmful effects [7,8].

There are several in vitro models mimicking the morphological and functional features of small intestine enterocytes that have been extensively used in nanoparticle toxicity studies. One example is the human colon adenocarcinoma Caco-2 cell line, which can undergo spontaneous differentiation and the formation of a cell monolayer [9].

While in vitro monolayer systems recapitulate essential aspects of gut barrier physiology, they provide limited information on more complex aspects, such as inflammation and immunity [10]. Neutrophils are key players in the immune response and the most abundant form of white blood cells, as well as part of the first line of defence against xenobiotics, including nanoparticles [11]. One of the major immune-regulatory factors implicated in AgNP-induced intestinal inflammation is, undoubtedly, oxidative stress, headed by the production of reactive species, which is mainly regulated by neutrophils [12]. Within this context, the direct interaction between AgNP and human neutrophils requires further studies [13,14,15,16].

The protection of the human organism against the harmful effects of reactive prooxidant species can occur endogenously, through the action of endogenous antioxidant enzymatic and non-enzymatic systems, or exogenously, through a variety of mechanisms effected by bioactive food components (e.g., polyphenols, polyunsaturated fatty acids and vitamins).

Flavonoids represent the most common group of plant polyphenols and are currently considered as an indispensable component in several nutraceutical, pharmaceutical, medicinal and cosmetic applications, essentially due to their recognized antioxidant and anti-inflammatory activities [17]. According to the existing data, an intake level of 1 mg of flavonoids results in a concentration of more than 40 µM in the intestinal lumen. Considering that, in Western populations, the estimated daily intake of flavonoids varies from 5 to 100 mg/day, it opens a window to explore the protective effect of these compounds at the GIT level [18]. To the best of our knowledge, there are only two papers suggesting a promising level of protection mediated by flavonoids, quercetin and kaempferol, against AgNP-induced intestinal cytotoxicity [18,19]. Accordingly, we consider that further attention should be given to the protective effects of dietary flavonoids against the pro-inflammatory effects of AgNP in the GIT.

**Figure 1:**
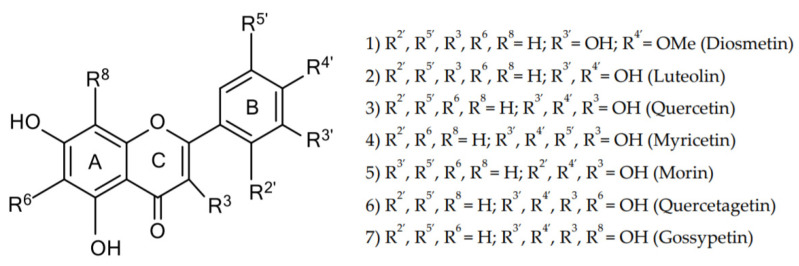
Chemical structures of the studied flavonoids.

In view of the unknown toxicological profile of AgNP with regard to the GIT, the hypothesis in this study was that AgNP may cause deleterious effects that may be prevented by flavonoids. To test this hypothesis, two in vitro systems were used: the Caco-2 model, representing human enterocytes, and freshly isolated human neutrophils as key players in intestinal immunity. Cells were exposed to polyethylenimine (PEI)-coated AgNP alone or concomitantly with a panel of structurally related flavonoids (Figure 1),and chosen based on their ability to modulate the inflammatory process [20,21,22,23,24]. Their interactions were studied.

## 2. Materials and Methods

### 2.1. Chemicals

All solvents and chemicals were of analytical grade. Silver nitrate (AgNO_3_), polyvinylpyrrolidone (PVP10, 10 kDa), branched polyethyleneimine (PEI, 25 kDa), diosmetin, dihydrorhodamine 123 (DHR), dimethyl sulfoxide (DMSO), 3-(4,5-dimethylthiazol-2-yl)-2,5-diphenyltetrazolium (MTT), Dulbecco′s phosphate-buffered saline pH 7.4 (DPBS), heat-inactivated fetal bovine serum (FBS), morin, nonessential amino acids solution (NEAA), *N*-(1-naphthyl)ethylenediamine dihydrochloride, phosphoric acid solution, quercetin, sodium nitrite (NaNO_2_), trypan blue solution, histopaque 1077, histopaque 1119, diphenyleneiodonium chloride (DPI), 1-(5-chloronaphthalene-1-sulfonyl)-1*H*-hexahydro-1,4-diazepine hydrochloride (ML-9), 3-[1-[3-(dimethylamino)propyl]-5-methoxy-1*H*-indol-3-yl]-4-(1*H*-indol-3-yl)-1*H*-pyrrole-2,5-dione (Gö6983), VAS2870 and sulfanilamide were purchased from Sigma-Aldrich (St. Louis, MO, USA). Dulbecco’s modified Eagle’s medium (DMEM) high glucose GlutaMAX™ supplement, trypsin:EDTA solution and 100× penicillin–streptomycin solution were purchased form ThermoFisher Scientific (Waltham, MA, USA). Luteolin and quercetagetin were purchased from Extrasynthese (Lyon Nord, France) and myricetin and gossypetin were purchased from Indofine chemical company (Hillsborough, NJ, USA). An FITC Annexin V Apoptosis Detection Kit was acquired from BD Pharmingen™ (Becton, NJ, USA). MilliQ (Millipore^®^ Burlington, MA, USA) deionized water was used in all synthetic procedures.

### 2.2. Methods

#### 2.2.1. Synthesis and Characterization of Silver Nanoparticles

##### Synthesis of Silver Nanoparticles

PEI-coated, PVP-stabilized AgNP were obtained by a wet-chemical procedure according to the method proposed by Sharonova et al. [25], with some modifications. First, PVP10 (8 wt.%) and PEI (2.5 wt.%) were dissolved in 240 mL of MilliQ water with magnetic stirring and reflux conditions. After heating the clear solution to 50 °C, 10 mL of AgNO_3_ solution (containing 1.6 wt.% of AgNO_3_) was added to the reactant solution and the temperature was further raised to 80 °C (in order to obtain AgNP 4 nm in diameter) or 100 °C (to prepare AgNP with a size of 19 nm). When the temperature reached 80 or 100 °C, the reaction was carried out for 5 h. After that, the obtained AgNP were separated from the reaction medium by centrifugation (at a relative centrifugal force maximum value of 23,000× *g* for 1 h) and washed with MilliQ water. Finally, PEI-coated AgNP were redispersed in MilliQ water.

##### Structural Characterization

X-ray diffraction was performed on an X-ray diffractometer (Philips PW1710, Panalytical, Brighton, UK) operated at 40 kV and 30 mA, and the spectrum was recorded by Cu Kα radiation with wavelength of λ = 1.54186 Å in the 2θ range of 20–80° with steps of 0.02° and 10 s/step.

##### Surface Chemistry Characterization

Surface functional groups of dried nanoparticles were analyzed by Fourier Transform Infrared (FTIR) Spectroscopy with a Thermo Nicolet Nexus spectrometer (Thermo Fisher Scientific, Madrid, Spain) using the attenuated total reflectance (ATR) method from 4000 to 400 cm^−1^.

##### Morphological characterization

The morphology and size of AgNP were determined by transmission electron microscopy (TEM), using a JEOL JEM-1011 microscope operating at 100 kV (JEOL, Tokyo, Japan).

##### Optical Characterization

Absorption spectra of both AgNP sizes were recorded on a Hewlett-Packard HP 8452A spectrophotometer (San Jose, CA, USA) in the UV−visible region.

#### 2.2.2. Human Intestinal Cells

##### Cell Culture and Treatments

The Caco-2 cell line, derived from a human colon adenocarcinoma (ATCC HTB-37, Rockville, MD, USA), was used between passages 30 and 50, and cultured in DMEM containing high glucose (25mM), sodium pyruvate (1 mM), glutaMAX™ (3.97 mM) supplemented with 10% heat-inactivated FBS, 10 × 10^3^ U/mL penicillin, 10 mg/mL streptomycin and 1% (*v*/*v*) nonessential amino acids (NEAA). Cells were grown in a 5% CO_2_ atmosphere at 37 °C. For each experiment, the cells were used when reaching 80% of confluence, which represented 3 to 5 days of differentiation. Then, the proliferating Caco-2 cells were treated 24 h after seeding.

##### Cell Viability Assays

MTT Assay

The MTT reduction assay was used to determine the cytotoxicity of PEI coated-AgNP of two sizes (4 and 19 nm) in the Caco-2 cell line. Briefly, 1.25 × 10^5^ cells were seeded in 96 well plates and were incubated for 24 h with 4 nm and 19 nm AgNP (5 to 50 μg/mL), or with flavonoids (3.13 to 50 μM). Non-cytotoxic concentrations of flavonoids (Table 1) were also tested together with AgNP in two sizes (4 and 19 nm) and concentrations (20 and 50 μg/mL). In addition, Caco-2 cells were exposed for 24 h to PEI (2.5–40 μg/mL) and AgNO_3_ (1.1–9 μg/mL) to rule out the cytotoxic effects of the coating (PEI), *per se*, and of the soluble silver ion, respectively. After the exposure period, the culture medium was replaced with fresh medium containing 0.5 mg/mL MTT and the cells were further incubated for 2 h. The resulting dark purple crystals of formazan were then dissolved in DMSO and the absorbance of the corresponding solution, which was directly proportional to the number of living cells, was measured in an automatic plate reader set at a test wavelength of 570 nm and a reference wavelength of 620 nm.

Annexin V Versus Propidium Iodide

The evaluation of cell death was analyzed by flow cytometry, following simultaneous staining with propidium iodide (PI) and Annexin V labeled with fluorescein isothiocyanate (FITC), according to a previously described method [13], using the Annexin V-FITC Apoptosis Detection Kit. Briefly, 5 × 10^5^ cells/well were seeded in 6 well plates and exposed to 20 μg/mL AgNP (4 and 19 nm) and/or to the flavonoids quercetin or quercetagetin (25 μM) for 24 h. Similarly, the cell death evaluation was performed for PEI (20 μg/mL) and AgNO_3_ (4.5 μg/mL) corresponding to the maximum PEI and Ag equivalent concentrations. After the 24 h exposure, cell supernatants were discarded, and cells were washed with DPBS buffer and detached from cells plates with trypsin:EDTA solution. Individualized cells were counted in a Neubauer chamber, and after DPBS wash, cells were resuspended at a density of 1 × 10^6^ cells/mL binding buffer provided by the kit. Subsequently, 1 × 10^5^ cells were transferred to suitable cytometry tubes, and Annexin V-FITC and PI were added. The mixture was incubated for 15 min and at least 10,000 events were immediately read in a BD Accuri™ C6 Plus Flow Cytometer (BD biosciences, Becton, Franklin Lakes, NJ, USA). The green fluorescence corresponding to Annexin-V conjugated with FITC was followed in channel 1 (FL1) and plotted as a histogram of FL1 staining. Fluorescence due to the PI incorporation was followed in channel 3 (FL3).

##### Measurement of Reactive Prooxidant Species

Oxidation of DHR

The AgNP-induced intracellular reactive oxygen species (ROS) production in Caco-2 cells was detected using the probe DHR. This is a cell-permeable non-fluorescent probe which, within the cells and in the presence of ROS, is oxidized to fluorescent rhodamine 123. Briefly, 5 × 10^5^ cells/well were seeded in 96-well plates and treated with AgNP (4 and 19 nm), in concentrations ranging from 5 to 30 µg/mL. DHR was added to the cell culture in a concentration of 5 µM, for the 24 h of exposure to the nanoparticles, at 37 °C, in a humidified 5% CO_2_ atmosphere. Fluorescence readings were taken at two different timepoints (3 h and 24 h) after AgNP treatment, in a microplate reader (λ_excitation_ = 507 ± 20 nm/λ_emission_ = 529 ± 20 nm).

Griess reaction

Nitrite measurement was based on the colorimetric detection of the reaction product of nitrite with naphthylethylenediamine dihydrochloride. The concentration of nitrite, which reflects nitric oxide (^•^NO) production, was measured in the cell-free supernatants collected from the Caco-2 cell line. Cell cultures were treated for 24 h with different concentrations of 4 nm and 19 nm AgNP (20 and 30 μg/mL), with or without flavonoids, PEI (20 and 40 μg/mL) or AgNO_3_ (4.5 and 9 μg/mL). After the incubation period, 150 μL of culture medium was collected and placed in 96-well plates. An equal volume of Griess reagent (0.2% naphthylethylenediamine dihydrochloride and 2% sulphanilamide in 5% phosphoric acid) was added, and the plates were incubated for 30 min, in the dark and at room temperature. The absorbance was measured at 570 nm using an automatic plate reader.

#### 2.2.3. Human Neutrophils

##### Isolation of Human Neutrophils

All patient-related procedures and protocols were performed in accordance with the Declaration of Helsinki and approved by the Ethics Committee of Centro Hospitalar do Porto. After written informed consent was obtained, venous blood from healthy human volunteers was collected by antecubital phlebotomy into vacuum tubes with K_3_EDTA. The isolation of human neutrophils was performed by the density gradient centrifugation method, as previously reported by our research group [26]. Cells were resuspended in Tris buffer (25 mM Tris, 1.26 mM CaCl_2_, 5.37 mM KCl, 0.81 mM MgSO_4_, 140 mM NaCl and 5.55 mM d-Glucose) and the cell viability and cell yield (number of cells/mL) were determined by the trypan blue exclusion method using a Neubauer chamber. Neutrophil suspensions (>98% cell viability) were kept on ice until use.

##### Cell Viability Assay

Isolated neutrophils (3 × 10^6^ cells/mL) were incubated with 4 nm and 19 nm AgNP (0–12.5 μg/mL) or with flavonoids (0–25 µM) for 1 h 30 min at 37 °C. The corresponding maximum PEI (10 μg/mL) and AgNO_3_ (3 μg/mL) equivalent concentrations of AgNP tested (12.5 μg/mL) were also tested.

Following exposure, cells were centrifuged (400× *g* at 20 °C, during 5 min) and the supernatant was discarded. The pellet was resuspended in DPBS solution and centrifuged (200*g* at 20 °C, during 5 min). After centrifugation, the pellet was resuspended with 100 μL propidium iodide solution (1 µg/mL) and incubated for 15 min in the dark. After this time, 400 μL of DPBS buffer was added and the fluorescence was measured in flow cytometer. To restrict the analysis to neutrophils only, a polygon gate was set according to their light scattering properties (in a forward vs. side scatter plot) excluding cell debris and other blood cells. Fluorescence signals for at least 10,000 cells were collected in logarithmic mode and the data were analyzed using C Flow (Accuri^®^) software (BD, Becton, Dickinson and Company, Franklin Lakes, NJ, USA). Fluorescence due to the PI incorporation was followed in channel 3 (FL3).

#### 2.2.4. Measurement of Neutrophil Oxidative Burst

The measurement of neutrophil oxidative burst was performed by monitoring the oxidation of DHR, by neutrophil-generated reactive species [13]. Neutrophils (3 × 10^6^ cells/mL) were incubated in a humidified incubator, at 37 °C, with 4 nm or 19 nm AgNP (0–10 µg/mL) alone or with flavonoids (0–25 µM) followed by DHR (10 µM) for 1 h 30 min. PEI (10 μg/mL) and AgNO_3_ (3 μg/mL) were also tested.

The NADPH oxidase inhibitors, DPI (10 µM) and VAS2870 (25 µM), and the protein kinase C (PKC) inhibitor, Gö6983 (1 µM), were also tested, by their concomitant addition with AgNP (10 µg/mL). The fluorescence was measured in a microplate reader (λ_excitation_ = 507 ± 20 nm/λ_emmission_ = 529 ± 20 nm).

#### 2.2.5. Statistical Analysis

The GraphPa d Prism 6 software (version 6.0, GraphPad software, San Diego, CA, USA) was used to calculate all the mean ± standard deviations of the mean (SEM), (from at least 4 individual experiments, performed in triplicate in each experiment). Statistical comparison between groups was estimated using the one-way analysis of variance (ANOVA), followed by the Bonferroni′s post hoc test. In all cases, *p*-values lower than 0.05 were considered as statistically significant.

## 3. Results

### 3.1. Characterization of Silver Nanoparticles

**Figure 2:**
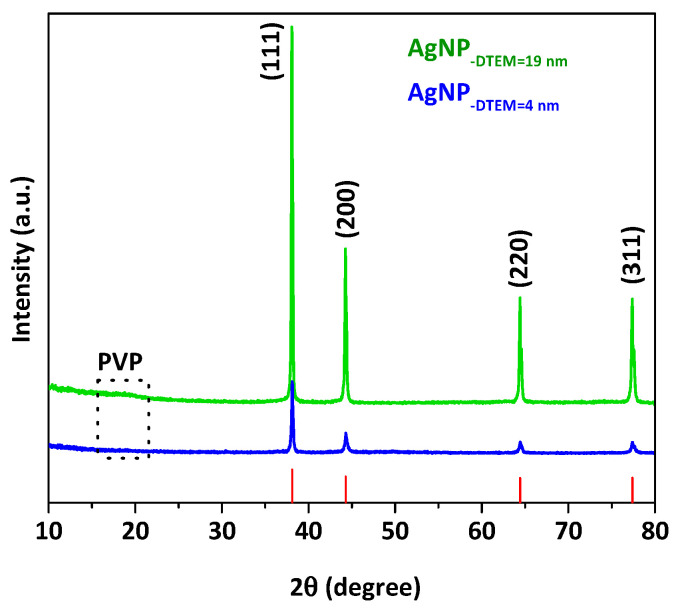
X-ray diffraction (XRD) patterns of AgNP with average diameters of 19 nm (green) and 4 nm (blue), compared to the XRD pattern of the Ag FCC crystal structure (JCPDS card No. 04-0783).

Figure 2 shows the diffractograms of both sizes of PEI-coated, PVP-stabilized AgNP, which display four characteristic peaks of the crystalline Ag phase, according to the face-centered cubic (FCC) crystal (JCPDS 04-0783) [27]. Additionally, a light broad band at low angles (2θ < 20°) is related to the presence of PVP [28].

**Figure 3:**
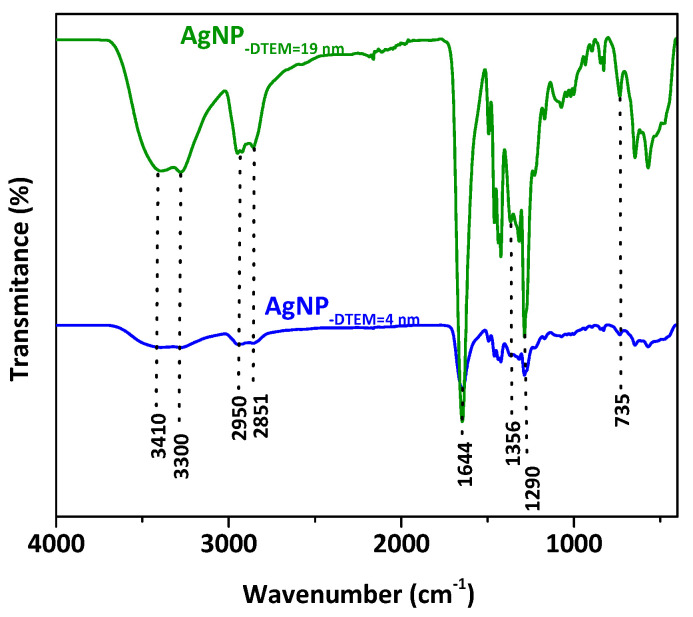
Fourier-transform infrared spectroscopy (FTIR) spectra of synthesized, PEI-coated, PVP-stabilized AgNP with average diameters of 19 nm (green) and 4 nm (blue), with the characteristic bands as evidence.

As shown in Figure 3, FTIR spectra of both samples show similar absorption bands, which is due to sharing the same composition of surfactants/stabilizing agents in the synthesis process. The double absorption bands located at 3410 and 3300 cm^−1^ are attributed to the O-H stretching vibration peak of PVP [28] and to the absorption peak of N-H stretching vibration of PEI [29], respectively. Moreover, two adsorption bands can be observed at 2950 and 2851 cm^−1^ that can be related to the asymmetric and symmetric CH_2_ stretching vibrations of PVP and PEI [28,29]. The peaks appearing at 1644, 1356 and 735 cm^−1^ are assigned to the C=O stretching vibration present in the amide group and the C-N (stretching and bending) vibrations modes of PVP [30], respectively. In addition, the peak appears at 1290 cm^−1^ provides the presence of the amine group of PEI [29] (N-H bending vibration).

**Figure 4:**
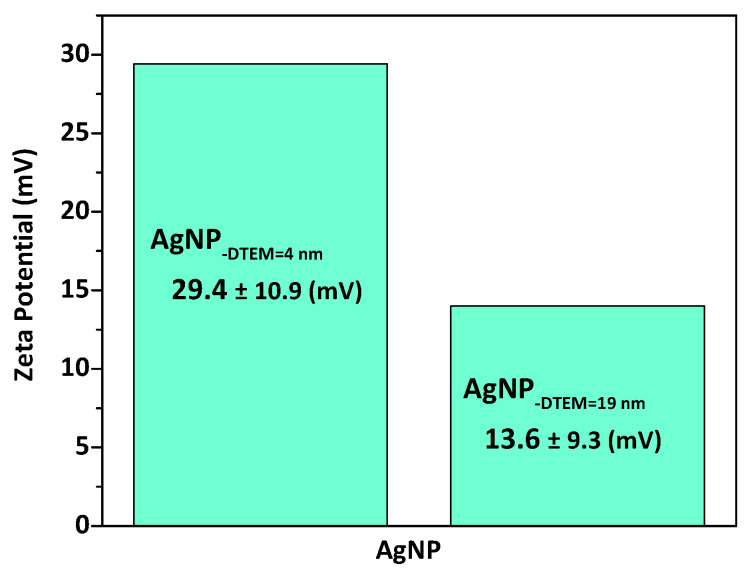
Zeta potential characterization of PEI-coated, PVP-stabilized AgNP performed in aqueous solution at neutral pH.

Additionally, in order to determine surface charge of the AgNP, electrophoretic mobilities were measured. Zeta potential measurements were performed by laser Doppler anemometry (LDA). LDA analyses were performed using a NanoZS^®^ (Malvern Instruments, Malvern, UK) at a neutral pH. Figure 4 shows the zeta potentials of both sizes of PEI-coated, PVP-stabilized AgNP, where the zeta potential increased with reduction in particle size, meaning more amine groups presented on the surfaces of smaller AgNP, which was due to a better coating of the polymer PEI on the entire nanoparticle [25].

**Figure 5:**
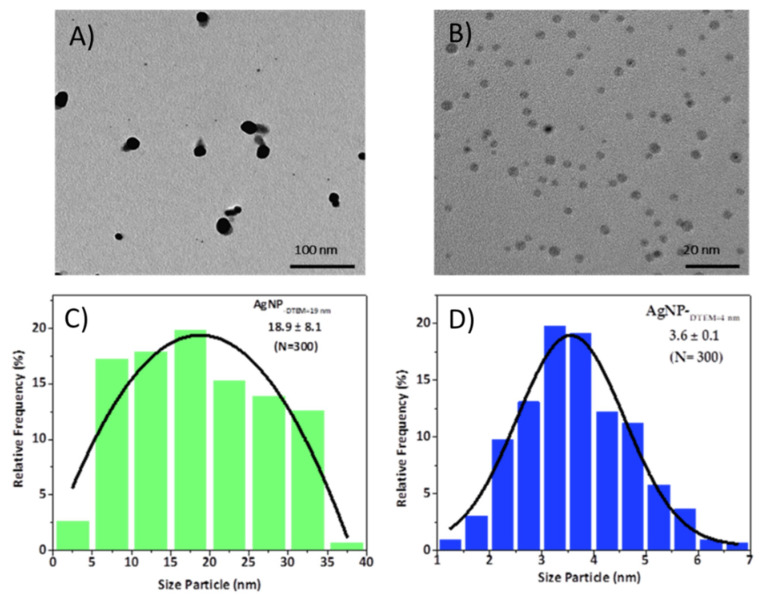
TEM micrographs and size distribution histograms of the PEI-coated, PVP-stabilized AgNP: 19 nm (**A**,**C**) and 4 nm (**B**,**D**). Size distribution was performed using Image J software (https://imagej.nih.gov/ij/download.html).

The morphology and size of AgNP (Figure 5) were also determined by TEM. The TEM images reveal that larger AgNP (Figure 5a) have quasi-spherical morphology with an agglomeration tendency and a non-homogeneous size distribution (Figure 5c) with averaged size around 19 nm. However, it can be clearly observed that smaller dispersed AgNP (Figure 5b) show a monodispersed spherical shape in the size range of 3.6 nm, with a narrow size distribution (calculated over 300 particles, Figure 5d).

**Figure 6:**
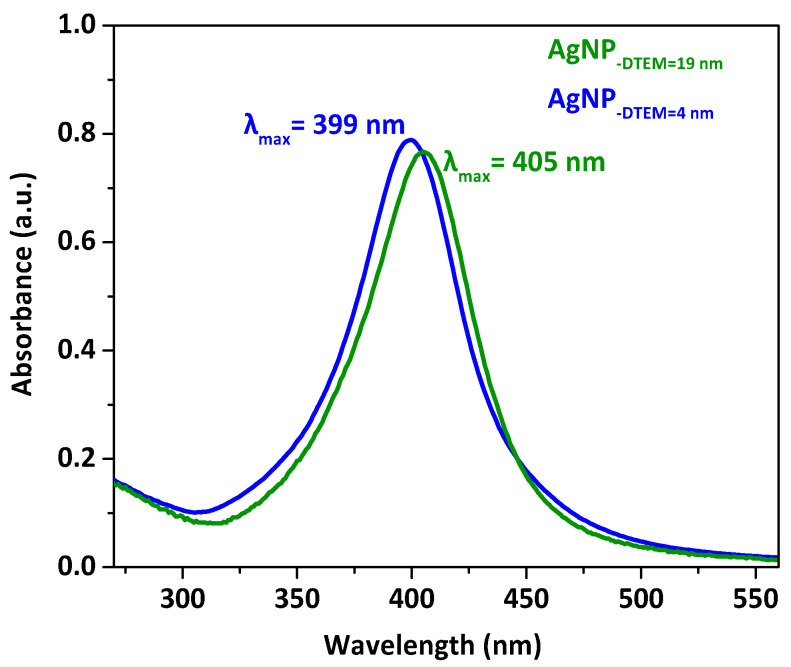
UV-Vis absorption spectra of PEI-coated, PVP-stabilized AgNP with average diameters of 19 nm (green) and 4 nm (blue), respectively.

As shown in Figure 6, smaller AgNP exhibit a plasmon resonance band near 400 nm, while the absorption maxima of larger AgNP shifted to longer wavelengths (located at 405 nm). These results were similar to previous work [31].

### 3.2. Intestinal Cells

#### 3.2.1. Evaluation of Cytotoxicity Induced by AgNP

**Figure 7:**
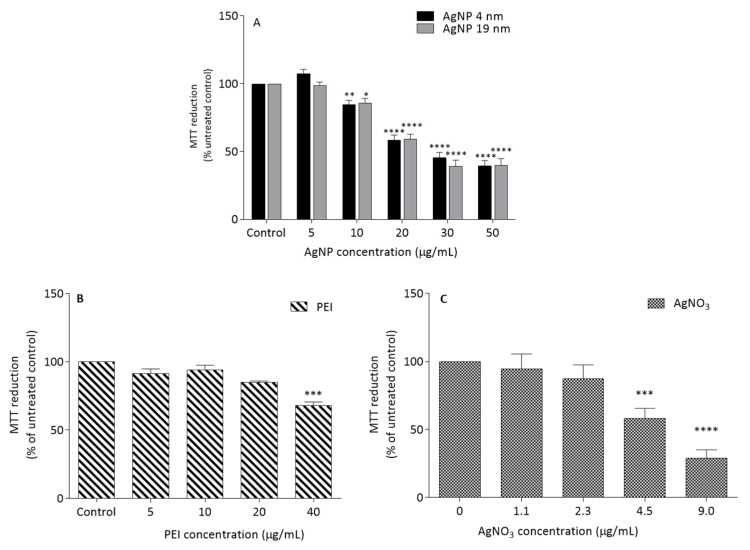
Effects of AgNP of 4 and 19 nm (**A**), PEI (**B**) and AgNO_3_ (**C**) in Caco-2 cell viability, after 24 h of exposure, measured by MTT reduction assay. * *p* < 0.05, ** *p* < 0.01, *** *p* < 0.001, **** *p* < 0.0001, when compared with the control (untreated cells). Values are presented as the means ± SEM (*n* ≥ 3).

MTT reduction assay was firstly used to determine the cytotoxicity of the AgNP in the Caco-2 cell line. AgNP induced cytotoxicity in a concentration-dependent manner (Figure 7A). Concentrations higher than 30 µg/mL of both AgNP sizes induced a cell viability reduction above 50%, when compared with the untreated control. Moreover, as shown in Figure 7B, only 40 μg/mL PEI, which corresponded to the maximum concentration of PEI obtained after dissolution of 50 μg/mL of 19 nm AgNP induced a decrease in cell viability below 30%. AgNO_3_ concentrations higher than 4.5 μg/mL, corresponding to the maximum silver ion concentration obtained with complete dissolution of 4 nm AgNP (30 μg/mL) or 19 nm AgNP (20 μg/mL), induced a cytotoxic effect above 50% (Figure 7C).

**Figure 8:**
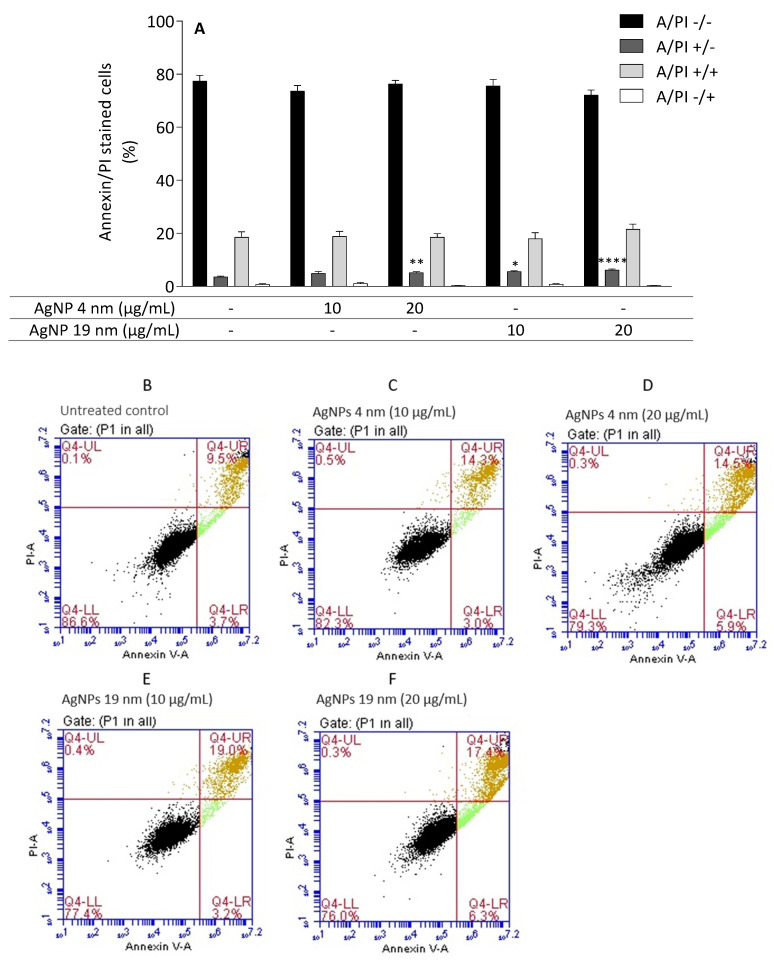
Apoptotic versus necrotic effects of AgNP in the Caco-2 cell line. Cells were incubated with 4 nm and 19 nm AgNP (10 and 20 µg/mL) for 24 h. Apoptotic and necrotic populations were quantified by flow cytometry with annexin V-FITC and PI. * *p* < 0.05, ** *p* < 0.01 and **** *p* < 0.001 when compared with the control (untreated cells) (**A**). Values are presented as the means ± SEM (*n* ≥ 5). Panels (**B**–**F**) correspond to representative flow cytometry plots of Annexin-V/PI binding assay [(annexin-V; *x*-axis)/PI (*y*-axis) (**B**)–Untreated control; (**C**,**D**)–AgNP 4 nm, 10 and 20 μg/mL; (**E**,**F**)–AgNP 19 nm, 10 and 20 μg/mL)].

Cell death was analysed by flow cytometry following staining with Annexin V-FITC and PI (Figure 8). For this purpose, the lowest AgNP concentrations that induced cytotoxic effects were chosen (10 and 20 µg/mL), as well as the corresponding PEI (20 μg/mL) and AgNO_3_ (4.5 μg/mL) concentrations. When compared to the control, the AgNP treatments increased the Annexin V+/PI– (early apoptotic) cell population (Figure 8A). Comparing nanoparticle size, 19 nm AgNP showed higher apoptotic effects compared to 4 nm AgNP, as evidenced by a significant increase in the early apoptotic cell population at lower AgNP concentrations. As an example, the concentration of 10 µg/mL AgNP induced an increase in the apoptotic cells only when 19 nm was used. At the tested concentrations and under the assay conditions, PEI and AgNO_3_ did not induce significant alterations in cell viability (data not shown).

#### 3.2.2. Protective Role of Flavonoids against AgNP-Induced Cytotoxic Effects on Intestinal Cells

As an initial step in the selection of flavonoid concentrations, the cytotoxicity of these compounds was evaluated in Caco-2 cells by the MTT assay. Within the range of non-cytotoxic concentrations tested, the highest non-cytotoxic concentration of each flavonoid was selected for subsequent studies (Table 1).

**Figure 9:**
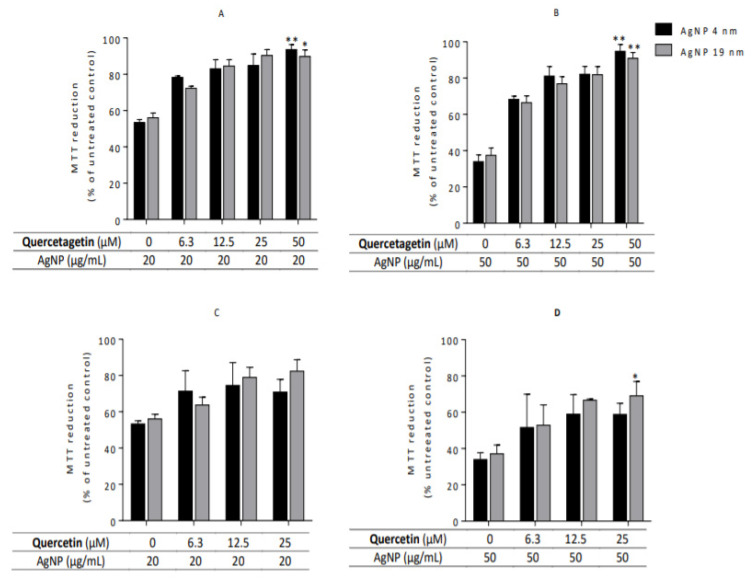
Effect of flavonoid concentration on AgNP-induced cytotoxicity in Caco-2 cells. After 24h co-incubation with cytotoxic AgNP concentrations and non-toxic flavonoid concentrations, cell viability was determined by MTT assay. Cells were incubated with 20 μg/mL AgNP [Subfigures (**A**,**C**)] and 50 μg/mL AgNP [Subfigures (**B**,**D**)]. MTT reduction percentages are normalized to untreated cells (100% MTT reduction). * *p* < 0.05, ** *p* < 0.01 when compared with AgNP-treated cells (no flavonoids). Values are presented as the mean ± SEM (*n* ≥ 3).

Among all the studied flavonoids, quercetagetin was the compound that most effectively protected against AgNP-induced cytotoxicity in Caco-2 cells, measured by the MTT assay (Figure 9A,B vs. Figure 9C,D). Two concentrations of AgNP were tested, 20 µg/mL and 50 µg/mL. The latter decreased cell viability by more than 50% when compared with the untreated cells. Quercetagetin (50 µM) prevented the cytotoxic effects of the two sizes of AgNP, even at high AgNP concentrations (Figure 9B), the protective effect being dependent on the tested concentration, independent of AgNP size. Quercetin, at non-toxic concentrations, partially prevented AgNP cytotoxicity (Figure 9C,D). The strongest protective effect was found for 25 µM quercetin in Caco-2 cells exposed to 19 nm AgNP. Nevertheless, an increase in the average values of cell viability was observed for all the tested quercetin concentrations.

**Figure 10:**
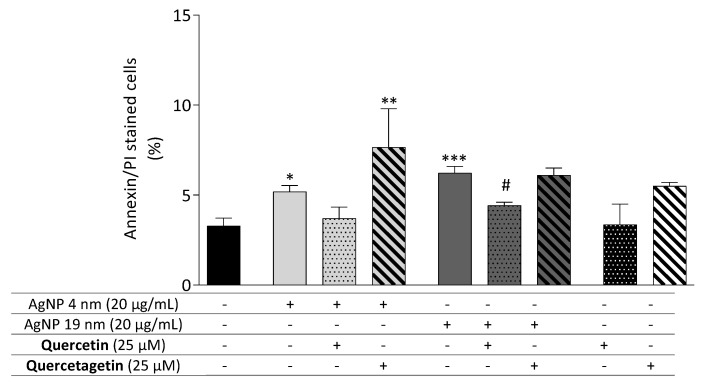
Effect of flavonoid co-incubation on apoptosis of Caco-2 cells exposed to an apoptotic concentration of AgNP. Cells were co-incubated for 24 h with 4 nm and 19 nm AgNP (20 µg/mL—apoptotic concentration) and flavonoids. Apoptotic and necrotic populations were quantified by flow cytometry with annexin V-FITC and PI. * *p* < 0.05, ** *p* < 0.01 and *** *p* < 0.001 when compared with the control (untreated cells). ^#^ *p* < 0.05 when compared with the respective AgNP-treated cells. Values are presented as the means ± SEM (*n* ≥ 5).

Moreover, the apoptosis-preventive effects of quercetin and quercetagetin were determined by flow cytometry analysis of annexin-V and PI staining. Quercetin (25 μM) reversed the pro-apoptotic effect of AgNP (Figure 10). Notably, quercetin reduced the percentage of AgNP (4 and 19 nm)-induced apoptotic cells to values that were not significantly different to those of untreated cells. Compared to the AgNP controls, quercetagetin, however, did not significantly decrease the apoptotic population when co-incubated with the respective AgNP. On the contrary, co-treatment with 4 nm AgNP (20 μg/mL) and quercetagetin increased the percentage of apoptotic cells.

#### 3.2.3. Measurement of Reactive Oxygen Species

The production of intracellular reactive oxygen species was measured by the fluorescent probe DHR. In the tested conditions, at two exposure time points, 3 and 24 h, none of the AgNP whether 4 or19 nm (0–30 μg/mL) induced the production of reactive species in Caco-2 cells. AgNP (19 nm) showed a slight increase in DHR oxidation; however, statistical significance was not reached (data not shown).

**Figure 11:**
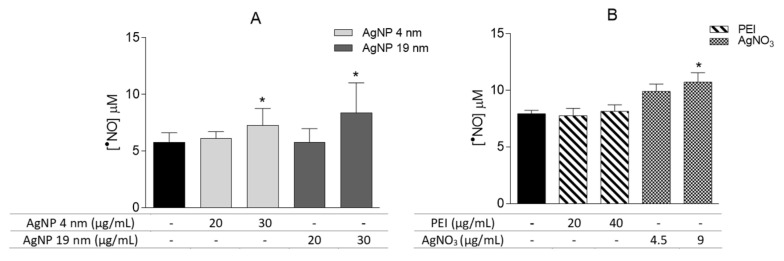
Effects of 4 nm (20 and 30 μg/mL) and 19 nm (20 and 30 μg/mL) AgNP. (**A**) and PEI and AgNO_3_ (**B**) on ^•^NO production, after 24 h of exposure, measured by Griess reaction. * *p* < 0.05, when compared with the control (untreated cells). Values are presented as the means ± SEM (*n* ≥ 5).

The effect of AgNP in Caco2-induced production of ^•^NO was analyzed by measuring accumulation of nitrite in the culture medium through the Griess reaction. As shown in Figure 11A, after 24 h, cells treated with both 4 nm and 19 nm AgNP (30 μg/mL) produced significant ^•^NO levels (7.3 ± 0.5 µM and 8.4 ± 0.9 µM, respectively) when compared with the untreated cells. Figure 11B shows that only 9 μg/mL AgNO_3_ induced a significant production of ^•^NO.

In order to understand if the flavonoids were able to prevent ^•^NO production induced by AgNP, they were all tested at their maximum non-cytotoxic concentrations. Under the tested conditions none of them were able to significantly prevent AgNP-induced ^•^NO production (data not shown).

### 3.3. Human Neutrophils

#### 3.3.1. Cytotoxicity of AgNP

**Figure 12:**
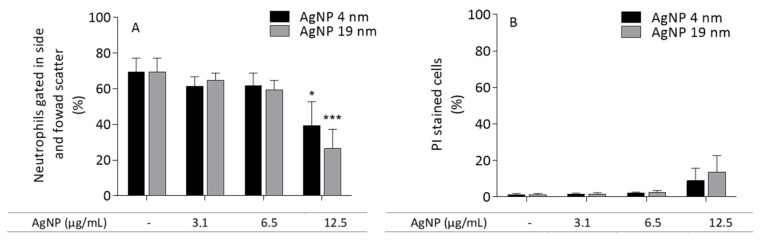
Flow cytometric analysis of human neutrophils incubated with 4- and 19 nm AgNP, using the PI staining method. (**A**,**B**) graphics summarize the results obtained in the neutrophils gated in side and forward scatter, and PI-stained cells, respectively. * *p* < 0.05 and *** *p* < 0.001 when compared with the control (untreated cells). Values are presented as the means ± SEM (*n* ≥ 4).

None of the AgNP concentrations tested (3.1, 6.3 and 12.5 µg/mL) resulted in a significant increase in PI-positive (necrotic/late apoptotic) cells. Nonetheless, a slight increase in the necrotic cell population was observed in cells incubated with 12.5 µg/mL AgNP, and this was associated with decreased cell numbers (Figure 12A,B). PEI and AgNO_3_ did not affect neutrophil viability in the tested experimental conditions (data not shown).

#### 3.3.2. Measurement of Reactive Prooxidant Species

**Figure 13:**
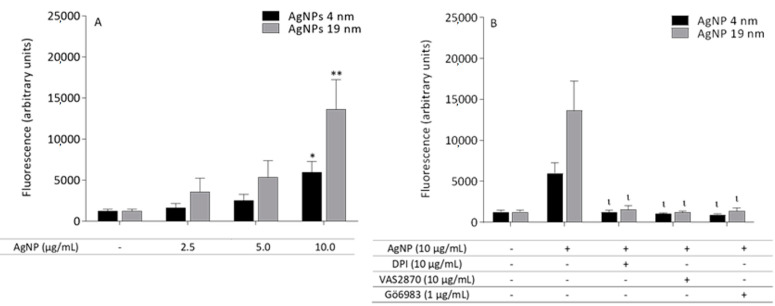
Effect of AgNP on neutrophil oxidative burst. Human neutrophils were exposed to 4nm and 19 nm AgNP (30 min) and the oxidative burst was determined upon incubation with DHR (**A**). For the AgNP concentration inducing neutrophil oxidative burst (10 μg/mL), PKC inhibitors were tested for effect prevention (**B**). * *p* < 0.05, ** *p* < 0.01, when compared with the untreated cells (**A**). ^ι^ *p* < 0.05, when compared with the cells treated with AgNP. Values are presented as the means ± SEM (*n* ≥ 6).

Human neutrophils treated with the two sizes of AgNP increased the production of reactive prooxidant species in a concentration-dependent manner. A higher reactivity for the 19 nm AgNP was found when compared with the 4 nm AgNP (Figure 13A). In human neutrophils, NADPH oxidase is the major enzyme system responsible for the production of ROS during the process of oxidative burst. Two known inhibitors of NADPH oxidase were used, namely, DPI and VAS2870, and both decreased DHR oxidation induced by 4nm and 19 nm AgNP (Figure 13B). PKC is directly involved in the activation of NADPH oxidase through phosphorylation of the subunit p47 [32]. When the specific PKC inhibitor Gö6983 was used, it also prevented the activation of neutrophil oxidative burst induced by AgNP (Figure 13B). PEI and AgNO_3_, in the tested experimental conditions, did not affect neutrophil oxidative burst (data not shown).

#### 3.3.3. Protective Role of Flavonoids against AgNP-Induced Neutrophil Oxidative Burst

The concentrations of flavonoids were chosen based in their non-cytotoxic effect in human neutrophils, measured by the Annexin-V/PI binding assay. According to Table 2, it is possible to conclude that all the tested flavonoids were effective inhibitors of AgNP-induced human neutrophil oxidative burst, except diosmetin, which at the maximum tested concentration (25 µM) did not reach the IC_50_. In general, the tested flavonoids more efficiently modulated the neutrophil oxidative burst induced by 4 nm AgNP, originating the lowest IC_50_. Among those, quercetagetin and quercetin were the most active flavonoids, presenting an IC_50_ < 1 µM. Interestingly, the scenario changed when AgNP of 19 nm were used, luteolin and morin being the most potent compounds, presenting an IC_50_ of 1.3 ± 0.2 µM and 1.5 ± 0.4 µM, respectively.

## 4. Discussion

In view of the increasing oral exposure to AgNP, and the knowledge that ingested AgNP are absorbed in the small intestine [33], it is imperative to extensively evaluate the potential toxic effects of AgNP on the GIT. Therefore, this work intends to encompass two of the leading-edge research hot topics: nanoparticle toxicity and positive health effects of food components, namely flavonoids. For that purpose, two sizes (4 and 19 nm) of PEI-coated AgNP were tested in two relevant cellular models. The first model consisted of Caco-2 cells, which are commonly used as a cell model of intestinal epithelial cells, and the second consisted of human neutrophils, an essential component of the innate immune system. Subsequently, the protective role of a panel of structurally related flavonoids was tested against the harmful effects of AgNP.

The concentrations of AgNP used in the present study can be attained in individuals following high repeated exposure or following acute accidental exposure to AgNP. As an example, using exposure data from an AgNP-manufacturing facility, 10 μg/mL of AgNP would approximately correspond to the total cellular deposition following 74 working weeks (8 h per day, 5 days per week) [34].

AgNP of both sizes induced alterations in Caco-2 cell viability, as measured by the MTT reduction assay. Our results demonstrated that concentrations above 10 µg/mL induced a significant reduction in cell viability, but only concentrations above 30 µg/mL of both sizes of AgNP (4 and 19 nm) induced a cell viability reduction higher than 50%. The observed toxicity with PEI and AgNO_3_ may have slightly contributed to the effect observed for nanoparticles. Nevertheless, it is accepted that AgNP are generally stable, slowly dissolving into ions on a time scale of several days [7].

A previous study showed that citrate-stabilized and PEI-stabilized AgNP have a limited silver ion release, i.e., the particles did not completely dissolve in an aqueous solution for up to 125 days. Transposing this to our study, the maximum 24 h exposure would not be enough to release the total amount of silver ions that would directly influence the cell viability.

To better understand whether AgNP-induced cell death occurred through necrosis or apoptosis, cells were analyzed by flow cytometry following simultaneous staining with Annexin V labeled with FITC and PI. In our experimental conditions, the treatment of Caco-2 cells with both sizes of AgNP resulted in an increase in Annexin V positive cells, representing an increase in apoptotic cells.

There are very few toxicological studies involving the exposure of Caco-2 cells to AgNP. In general, those studies show that AgNP are toxic to these cells. Exposure of proliferating Caco-2 cells to small peptide l-cysteine/l-lysine/l-lysine-coated AgNP of 20 and 40 nm, decreased the adherence capacity and induced cytotoxicity. Nevertheless, the authors reported that smaller nanoparticles (20 nm) exhibited higher toxicity than the 40 nm particles. The toxic pathway was suggested to involve necrosis rather than apoptosis [4]. Later, the same research group studied surfactant-coated AgNP and also reported a cytotoxic effect in Caco-2 cells not related to apoptosis [5]. Martirosyan and co-workers [18,19] also reported the cytotoxic effect of 20 nm of AgNP (15–90 µg/mL) in Caco-2 cells, and found an EC_50_ of ca. 40 μg/mL (MTT assay). Song et al. [35] demonstrated the inhibitory effect of AgNP (0 to 200 µg/mL) on Caco-2 cell proliferation, as well as their dose-dependent cytotoxic effect, using the CCK-8 assay. Similarly, Mao and co-workers [36] found a dose-dependent cytotoxic effect of AgNP (35–40 nm) at concentrations ranging from 0.5 to 3 mM, as measured by MTT assay, which was maintained under acidic gastric fluid environment conditions. In contrast, Bouwmeester et al. [8] tested 20, 34, 61 and 110 nm AgNP for 24 h in proliferating Caco-2 cells, and did not detect any influence on the cells’ metabolic activity up to 50 µg/mL. It is known that several parameters could be responsible for AgNP toxicity, including size, surface area, surface chemistry, water and liquid solubility and coagulation or aggregation state [4]. Nonetheless, it seems consensual that AgNP reduce intestinal cell viability in a dose-dependent manner.

It has been established that the inflammatory process in the intestine is closely associated with the overproduction of both ROS and reactive nitrogen species (RNS), which may result in detrimental effects for the host, in a process commonly known as oxidative stress, which in the last instance could induce cell death [37].

Taking this into account, DHR, a lipophilic fluorescent probe that readily permeates cell membranes, was used to detect H_2_O_2_ and HOCl. In our experimental conditions, it was not possible to detect a significant ROS production from 3 h to 24 h of incubation. Interestingly, the detection of ^•^NO through the Griess reaction showed that AgNP of both sizes induced the production of RNS. Regarding the production of ROS and RNS, the information found in literature is, once again, contradictory, depending on the type and size of the studied AgNP. Using the probe 2′,7′-dichlorofluorescin diacetate, some authors reported no production of reactive prooxidant species after treatment with ≤20 µg/mL of peptide-AgNP [4] or AgNP-dispersant [18,19], while other authors reported a probe oxidation by naked, citrate and PVP coated-AgNP (23, 24 and 30 nm) at low concentrations (0.7 µg/mL) [38]. Martirosyan et al. [19] also used Griess assay and reported that AgNP <20 nm and <20 µg/mL also induced the production of nitrite and nitrate, an indirect measure of ^•^NO production.

The intestinal mucosa displays, even under normal conditions, a state of “basal inflammation”, manifested by the presence of a large number of different immune cells. All of these cells coexist and act in perfect equilibrium, conferring both tolerance and protection to the gut. When the intestine suffers a significant pro-inflammatory stimulus, a disruption of the epithelial-cell barrier occurs, resulting in an increase in intestinal permeability and infiltration of immune cells, namely neutrophils [37]. One of the main mechanisms of defense of neutrophils against foreign bodies, such as nanoparticles, occur through two concurrent events in the phagolysosome of stimulated neutrophils: one is oxygen-dependent, known as “oxidative burst”, through the formation of ROS and RNS, and the other is oxygen-independent, consisting of the release of granular enzymatic or antimicrobial protein content [39]. The neutrophil oxidative burst initiates following a contact with an external stimulus, with subsequent activation of phospholipase C and the hydrolysis of phosphatidylinositol 4, 5-bisphosphate to generate diacylglycerol and inositol trisphosphate, which subsequently mobilize intracellular calcium. These events result in PKC activation, which is responsible for the phosphorylation of one of the subunits of the NADPH oxidase, p47, and ultimately mediates the production of reactive species in a cascade form [40].

Therefore, disclosing whether AgNP induce neutrophil oxidative burst became an appealing question. Our results clearly show that AgNP activate human neutrophils, inducing the production of reactive species in a size-dependent manner, since AgNP of 19 nm originate a higher oxidation of the probe when compared with AgNP of 4 nm. The information available in the literature about the effects of AgNP in the human neutrophil oxidative burst is scarce, lacking any reference to the use of PEI-coated AgNP. Our group [13,16] has previously shown that polyvinylpyrrolidone (PVP) and citrate-coated AgNP of 5 and 10 nm induced a greater production of reactive species in these cells. This production was higher when the smaller and citrate-coated AgNP were used. Interestingly, at concentrations ≤12.5 µg/mL, neither PVP- nor citrate-AgNP (5, 10 and 50 nm) induced DHR oxidation. These results contrast with those obtained in the present work, where significant activation was found with AgNP at 10 µg/mL. Once again, the different sizes and coating agents could justify the dissimilar results.

NADPH oxidase is the major enzyme system responsible for producing human neutrophil oxidative burst. DPI and VAS2870 were the NAPH oxidase inhibitors used in this study, the latter being a selective inhibitor of NOX-2 [41], which is the NADPH oxidase present in neutrophils [39]. Both inhibitors reduced the production of reactive prooxidant species induced by both AgNP sizes. Similarly, the broad-spectrum PKC inhibitor also reduced the neutrophil oxidative burst induced by 4 and 19 nm AgNP. These results suggest that AgNP induced an activation of PKC with subsequent stimulation of NAPH oxidase. This seems to be the common mode of activation of human neutrophil oxidative burst by AgNP, independent of the size and coating agent used [13,16].

Considering the potential antioxidant and anti-inflammatory activity of flavonoids, namely as modulators of neutrophil oxidative burst, our intention was to disclose the potential protective role of flavonoids against the harmful effects of AgNP. The number and type of flavonoids ingested as well as the levels detected in the human body vary due to several factors, such as the diet of each individual to the cooking and food processing methods. The average consumption of flavonoids ranges from 5 to 100 mg/day, depending on diets and habits, quercetin being the major representative (68–73%) [42,43].

Our results unveil a promising effect of widely consumed and easily accessible dietary flavonoids against AgNP pro-inflammatory effects. Using the intestinal cells, it was shown that among the tested flavonoids, quercetin and quercetagetin significantly reduced the cell death induced by AgNP of 4 and 19 nm, as measured by MTT and Annexin-V/PI assays. These results were corroborated by Martirosyan et al. [18], since they studied the effect of quercetin, kaempferol and resveratrol (10 and 50 µM) in Caco-2 cells treated with AgNP < 20 nm (30–90 μg/mL) and concluded that quercetin was the compound that most effectively reversed the loss of viability induced by AgNP. Later, the same research group reinforced that quercetin (50 µM) successfully reverted the toxicity induced by AgNP (≥45 μg/mL). Surprisingly, this protection does not seem to be mediated via a decrease in ^•^NO production, since neither quercetin nor quercetagetin prevented AgNP-induced ^•^NO production.

Promising results from the same flavonoids were also obtained in human neutrophils, namely in the inhibition of AgNP-induced neutrophil oxidative burst by flavonoids. In general, all the studied flavonoids were able to hinder the production of reactive species by neutrophils treated with AgNP of 4 and 19 nm, showing IC_50_ < 5 µM. Diosmetin was the exception, since it was not possible to achieve the IC_50_ at the higher tested concentration (25 µM). As this flavonoid was the only one that presented an -OMe group, this suggests that the methoxy substituent did not favor the intended activity.

Scrutinizing the obtained results, quercetagetin followed by quercetin were, again, the most active flavonoids (IC_50_ < 1 µM), when 4 nm AgNP were used. Interestingly, the IC_50_ increased by 6 and 3 times, respectively, when neutrophils were treated with 19 nm AgNP. This rise was reasonable, since 19 nm AgNP induced a higher production of reactive species, which may have required higher concentrations of flavonoids to inhibit that production. Considering the chemical structure of quercetin and quercetagetin, the presence of the catechol group in the B ring, as well as the presence of the -OH group at position 3 of the C ring, seems to be favorable for the inhibition of 4 nm AgNP-induced oxidative burst. This structural requirement changed slightly when 19 nm AgNP were used, the most effective flavonoids in this case being luteolin, followed by morin. Nonetheless, the catechol group was present in the luteolin scaffold and the -OH group at position 3 of the C-ring was present in morin.

The relevance of the B-ring substitution, namely the presence of a catechol group and the presence of an -OH group at position 3 of the C-ring in the inhibition of human neutrophil oxidative burst corroborated previous findings [20,24]. These studies used phorbol-12-myristate-13-acetate (PMA) as inflammatory stimulus in order to activate neutrophils. It is currently accepted that PMA activates the NADPH-oxidase through redistribution of PKC and phosphorylation of several proteins, including the cytosolic NADPH-oxidase subunit p47 [44]. As demonstrated, this was the mechanism by which AgNP induced neutrophil oxidative burst. Therefore, this justifies the fact that the flavonoids that were active in the modulation of PMA-stimulated neutrophils may be effective in oxidative burst reduction induced by AgNP.

## 5. Conclusions

This study showed that PEI-coated AgNP of 4 and 19 nm may alter the intestinal environment through induction of apoptosis with concomitant production of ^∙^NO, irrespective of the size used, and also through the stimulation of human neutrophil oxidative burst, being more pronounced in the case of 19 nm AgNP. It also showed that AgNP induce the production of reactive species in human neutrophils via PKC activation, with subsequent assembly of NADPH oxidase subunits, resulting in the production of reactive species.

We also found that dietary flavonoids, commonly consumed in our diet, protect the GIT from the deleterious pro-inflammatory effects of AgNP. It revealed that quercetin and quercetagetin were the most promising flavonoids, being able to prevent the harmful effects of AgNP in intestinal cells as well as in human neutrophils. Our results suggest that the presence of the catechol group in the B ring as well as the presence of the -OH group at position 3 of the C ring is favorable for the protective effect of flavonoids.

## Figures and Tables

**Table 1:** Maximum non-cytotoxic flavonoid concentrations in Caco-2 cells, as determined by MTT assay.

Compound	Selected Maximum Concentration (μM)
Diosmetin	12.5
Luteolin	12.5
Quercetin	25.0
Myricetin	50.0
Morin	50.0
Quercetagetin	50.0
Gossypetin	50.0

**Table 2:** Structures of the studied flavonoids and AgNP (4 and 19 nm)-induced inhibition in human neutrophils, assessed by DHR (IC_50_ μM, mean ± SEM).

Compound	Structure	R^2′^	R^3′^	R^4′^	R^5′^	R^3^	R^6^	R^8^	IC_50_ μM (Mean ± SEM)	
									AgNP 4 nm	AgNP 19 nm
Diosmetin	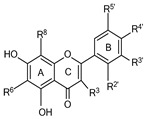	H	OH	OMe	H	H	H	H	37 ± 3 ^*^	37 ± 7 ^*^
Luteolin		H	OH	OH	H	H	H	H	1.3 ± 0.2	1.3 ± 0.2
Quercetin		H	OH	OH	H	OH	H	H	0.94 ± 0.09	3.1 ± 0.6
Myricetin		H	OH	OH	OH	OH	H	H	2.3 ± 0.1	3.1 ± 0.2
Morin		OH	H	OH	H	OH	H	H	1.4 ± 0.4	1.5 ± 0.4
Quercetagetin		H	OH	OH	H	OH	OH	H	0.53 ± 0.06	3.0 ± 0.2
Gossypetin		H	OH	OH	H	OH	H	OH	2.1 ± 0.3	4.5 ± 1.2

* Percentage of inhibition at the highest tested concentration, 25 μM.

## Data Availability

The data presented in this study are available on request from the corresponding author.

## Sample Availability

Samples of the tested compounds namely flavonoids andAgNP) are available from the authors.
